# The Impact of Psychological Capital and Social Capital on Residents’ Mental Health and Happiness During COVID-19: Evidence From China

**DOI:** 10.3389/fpsyg.2022.962373

**Published:** 2022-07-18

**Authors:** Xincheng Zhao, Qian Liu, Shan Zhang, Tinghua Li, Bin Hu

**Affiliations:** ^1^School of Economics and Management, Guangzhou City Construction College, Guangzhou, China; ^2^Faculty of Finance, City University of Macau, Macau, China; ^3^Faculty of Business, City University of Macau, Macau, China; ^4^School of Logistics, Guangdong Mechanical and Electrical Polytechnic, Guangzhou, China; ^5^Liberal Arts College, Changsha Normal University, Changsha, China

**Keywords:** psychological capital, social capital, mental health, happiness, ordered probit, SEM

## Abstract

**Objective:**

This paper studies the mediating and interactive effects of social capital on psychological capital and the feeling of happiness from the impact of COVID-19. Since its emergence, the COVID-19 pandemic has taken a toll on people’s mental health and affected their hopes for the future. Lifestyle and economic conditions have also been affected and have subsequently impacted people’s sense of confidence in life. This could increase the likelihood of many people developing mental health issues, such as anxiety or depression. Therefore, it is vital to study the influence of psychological capital and social capital on people’s subjective psychology and happiness experiences.

**Materials and Methods:**

Using an ordered probit model, this paper studied the independent influence and interaction between psychological capital and social capital on people’s happiness. The ordered probit model was chosen because subjective well-being (SWB) is an ordered variable. We further used structural equation modeling (SEM) to study the mediating effects of social capital on psychological capital and happiness.

**Results:**

The regression results showed that both psychological capital and social capital were significantly positively correlated with happiness when controlling for other factors. In addition, psychological and social capital significantly interacted, in which the psychological capital promotes the effect of social capital on happiness. Moreover, the effect of psychological capital on happiness was greater than that of social capital, demonstrating that happiness is more greatly influenced by subjective psychological experience. The interaction coefficient of psychological and social capital was also significant, showing that the two have mutually reinforcing effects on happiness. Finally, health, income class, real estate, stranger trust, age, and urban household registration had significant positive effects on happiness, while the view of money, being female, education had a negative relationship with happiness. The SEM results showed that the mediating effect of psychological capital on happiness was partly transmitted through social capital: the total effect of psychological capital on happiness was highly significant (*p* < 0.0001), as was the total effect of social capital on happiness (*p* < 0.0001); however, the coefficient for psychological capital was greater than that for social capital. Through heterogeneity analysis, we found that the relationship between psychological capital, social capital, and happiness was significantly positive in each sub-sample group. There was also a significant interaction between psychological and social capital for men, women, urban and rural residents, and higher education background sample groups. However, the interaction was not significant in the sample group without higher education. In addition, the relationship between the happiness of rural residents and their educational background and gender was not significant.

**Conclusion:**

We found that psychological and social capital have significant positive relationships and effects on happiness. Psychological capital demonstrated both direct and indirect influences on happiness, and further strengthens the influence of social capital on happiness. These results support a scheme to emphasize psychological support during the COVID-19 pandemic period to enhance the mental health of citizens.

## Background

The COVID-19 pandemic has had a significant impact on people’s psychological and subjective well-being (SWB), deeply impacted the global economy, and profoundly changed people’s lifestyles and social capital. In the process of combatting the pandemic, people were instructed to maintain social distancing, meaning that residents were now compelled to stay and work from home, which affected the quality and function of social networks. The combination of COVID-19 and anti-globalization has led to a global macroeconomic contraction, which has changed people’s perception of life. A greater number of citizens suffer from mental disorders, such as anxiety or depression. Therefore, it is urgent to study the influence of psychological and social capital on people’s SWB. While research in this area is rapidly evolving, several questions remain to be answered:

1. Does psychological capital promote the impact of social capital on happiness under COVID-19? Alternatively, at the technical level of research, do psychological capital and social capital have an interaction effect, and is this effect heterogeneous?
2. Does psychological capital have an indirect effect on happiness through social capital and, if so, to what extent? Alternatively, does social capital mediate the relationship between psychological capital and happiness?
3. What is the greater overall effect of psychological capital or social capital on happiness?

Such questions have significant theoretical and practical utility. Research has investigated SWB and happiness from multiple perspectives and fields across philosophy, psychology, sociology, ethics, and economics, finding that it is a largely heterogeneous construct. An important representative view in the field of happiness research suggests that an individual’s subjective happiness is measured by their characteristics in the environment. SWB, therefore, reflects the social functions of individuals and whether they adapt to the environment (Diener et al., 1999). This view has been adopted by many later researchers, who define happiness based on an individual’s subjective judgment as SWB; that is, SWB refers to the degree to which an individual feels contentment in his or her life (Diener, 2000).

Psychological capital comes from the field of positive psychology, especially positive organizational behavior, and has been widely referred to as PsyCap (Luthans and Youssef-Morgan, 2017). According to some researchers, psychological capital is a measurable and developable positive psychological ability (Luthans and Youssef, 2004) and suggest that it refers to a state of individuals in which they positively grow and develop. Psychological capital includes four dimensions: efficacy, hope, optimism, and tenacity (Ma et al., 2015; Mao and Tang, 2015). Psychological capital is also closely related to residents’ happiness (Ma et al., 2015) and is an important factor in promoting individual growth and development through the process of motivation stimulation (Wu et al., 2012). The concept also has a strong, direct, and positive relationship with happiness (Rabenu et al., 2016). Quantitative studies have shown that happiness is related to an individual’s internal psychological resources, particularly as it relates to hope and optimism for the future (Kun and Gadanecz, 2019). It is also generally believed that individuals with a higher level of psychological capital have higher SWB (Mao and Tang, 2015; Meng and Han, 2015).

Social networks are established based on people’s basic needs for relationships, a sense of belonging, and communication and social relationships (Deci and Ryan, 1985; Burroughs and Eby, 1998). These basic social relationship networks and rules are defined as social capital (Putnam and Leonardi, 1994), the concept of which builds on the work of previous researchers (Frey and Stutzer, 2002; Helliwell and Putnam, 2004; Crossley and Langdridge, 2005). Compared with traditional social capital based on blood relationships, a work-based social network has become an important aspect of the modern social capital of Chinese residents (Li and Zhu, 2014). The influence of social networks on personal happiness is complex, and positive social interactions can significantly improve personal happiness (Diener, 2009), or are occasionally negatively correlated (Bartolini et al., 2008; Sarracino, 2012). Through social networks, individuals can gain advantages in employment, income, and additional economic benefits (Powdthavee, 2008). Studies have further shown that single people tend to have a higher level of social capital, which is positively correlated with happiness (Kislev, 2020). Further work has found that trust, interaction, and social participation are positively correlated with happiness (Tsuruta et al., 2019).

Fukushima et al. (2021) conducted a stratified study on the moderating effects of individual social and psychological capital and found that the psychological connection of happiness was regulated by social capital at the community level, while social capital at the individual level had no significant moderating effects. In addition, Oshio (2016) further found that social capital at the individual level had a mediating effect on the impact of social capital at the community level on happiness. Further work has found that female managers with children rely on psychological capital to reduce the negative impact of work-family conflict on happiness (Machín-Rincón et al., 2020), and it has also been shown that psychological capital may indirectly affect individual behavior and state through intermediate variables (Vatan, 2015).

From the above summary, it is clear that the relationship between psychological and social capital and happiness has been extensively investigated, while other studies have focused more on mediating the effects of capital on happiness. However, the mediating effect of social capital on psychological capital and happiness is still unknown. In this study, we empirically analyze the mediating effects of social capital, the interaction between psychological and social capital, and the total effect of psychological and social capital on happiness.

## Data Source and Model Setting

### Data Source

The data used in this study were obtained from the China Family Panel Studies (CFPS) of the Institute of Social Science Survey (ISSS) of Peking University. Since the latest data are not available, the most recent observations are from 2018. The database contains data from questionnaires pertaining to individual and community-level, family member and household economics, self-reports, and parental reports on children. In this study, we solely focus on self-report and household economics data. Due to missing data, we obtained 28,184 valid samples after cleaning.

### Variable Selection

We selected the following variables for analysis:

*Happiness:* This data were obtained from the question “How happy do you feel you are?” Responses were assigned values from 0 (low) to 10 (high), in which 10 indicates the highest level of happiness.

*Psychological Capital*: referred to as PsyCap, this construct refers to the general core psychological ability of an individual. We, therefore, extracted responses to the question “Please rate your confidence level for your future.” Answers to the question ranged from 0 to 5, with higher ratings representing higher confidence levels.

*Social capital*: Oshio (2016) analyzed four types of social capital—trust in neighbors, connection with neighbors, bond, and bridge. For residents in a typical “guanxi” society in China, the evaluation of “popularity relations with people” integrates the social capital at individual and regional (or community) levels, neighborhood relations, and social networks. We, therefore, conceptualized this construct based on the popularity relationship score, in which 0 represents the lowest level and 10 represents the highest level.

We also selected the following control variables:

*Life satisfaction* refers to the extent of satisfaction regarding one’s life, with higher scores reflecting higher life satisfaction. The *health* variable reflects the self-assessment of respondents of their own health, and after re-coding, higher scores indicated better health status.

*An income class* reflects income status and larger values indicate a higher income class. Crucially, we did not use the absolute value of residents’ income level of the index to measure income due to heterogeneous differences across levels of income in China, and so the absolute income level in different regions cannot be horizontally compared. We, therefore, used the relative index to adjust for this disparity.

Additionally, we selected gender, age, property ownership, trust in others, attitudes toward money, educational background, and type of residence (urban or rural) as control variables.

### Model Settings

#### Ordered Probit Model

The ordered probit model assumes that each respondent has a personal real potential state of happiness Y, but that its real state is unmeasurable. Each respondent evaluated and assigned values according to their subjective happiness. We could only obtain the subjective ratings of the respondents’ happiness attitudes. In the following session, we assume that the Y* of resident is a linear function of the explanatory variable X: Y* =X ⋅β +ε, where β represents the coefficient vector, and the residuals follow a standard normal distribution. We can define *C*_*k*_ as the k segmented points of Y*. Respondents’ subjective happiness level was obtained based on the relative size of the real value of the Y* and k segmented points. Specifically, if an individual’s real happiness level meets Y* ≥ *C*_9_, an individual chooses Y = 10; if *C*_5_ ≤ Y* ≤ *C*_6_, an individual chooses Y = 6, and so on. Therefore, the following regression model can be set:


(1) $$\mathrm{Pr}\left(Happi_{i}=k\right)=\text{Pr}(c_{k-1}<\alpha_{1}Psych_{i}+\alpha_{2}Socia_{i}+\alpha_{3}\,P\,s\,y\,c\,h\,\_\,S\,o\,c\,i\,a_{i}+\alpha_{4}\,C\,t\,r\,s_{i}+\varepsilon_{i}\leq c_{k}$$

Where, *Ctrs*_*i*_ is the control variable. In addition, the marginal effect of each independent variable on the dependent variable can be calculated by the following equation:


(2) $$\frac{\partial \text{Pr}\left(Happi_{i}=k\right)}{\partial X_{j}}=\left[\phi^{\prime }\,\left({\hat{c}}_{k-1}-X\,\hat{\beta }\right)-\phi^{\prime }\,\left({\hat{c}}_{k}-X\,\hat{\beta }\right)\right]\cdot {\hat{\beta }}_{j}$$

In equation (1), *k* = 0,1,2⋯⋯10, corresponds, respectively, to the different scores of subjective happiness. In equation (2), ϕ is the standard normal distribution cumulative function and ${\hat{c}}_{k}$ and $\hat{\beta }$ are the parameter estimate values obtained from the regression equation.

#### Structural Equation Model

Structural equation modeling (SEM) was used to estimate the mediating effects of social capital and test whether these effects were significant. This approach also allows the investigation of direct, indirect, and total effects of psychological capital and social capital on happiness, as well as a measure of which variable has a greater impact on an outcome variable.

## Empirical Analysis

### Descriptive Statistics

Descriptive statistics for each variable are presented in Table 1, while the statistics of the explanatory variables are shown in Table 2. Most of the samples had a subjective happiness score of 5 or more, and 58.44% of the samples reached 8.8 or more, indicating an overall high level of residents’ happiness. For psychological capital, the proportion of residents with great confidence in the future reached 44.22%, and those with relatively high confidence were 30.88%. The statistics of the social capital variables do not show any particular trend in the popularity relationship score, as the most popular residents accounted for 15.76% and most scores were in the middle/upper range (value range: 5–8).

**TABLE 1:** Descriptive statistics.

Variable	Symbol	Definition	# of Obs	Mean	Std. Dev.	Min	Max
Residents’ subjective well-being (SWB)	Happi	Happiness from low to high was assigned from 0 to 10 points, with 10 points for the highest	28,184	7.476	2.175	0	10
Psychological capital	PsyCap	The value from low to high was assigned from 1 to 5, larger score means a higher confidence level	28,184	4.134	0.958	1	5
Social capital	SocCap	The value ranges from 0 to10, 0 represents the lowest and 10 represents the highest	28,184	7.132	1.956	0	10
Health	Healt	1. Unhealthy; 2. General; 3. Relatively healthy; 4. Very healthy; 5. Super healthy	28,184	3.052	1.215	1	5
Income class	InCla	Income level in local, larger score means higher income class	28,184	2.912	1.074	1	5
Sex	Male	Male = 1, Female = 0	28,184	0.5	0.5	0	1
Property ownership	House	Yes (House = 1) or Not (House = 0) owned property	28184	0.357	0.479	0	1
Trust in others	Trust	Most people can be trusted = 1; be careful to trust others = 0	28,184	0.554	0.497	0	1
Attitudes toward money	Money	The importance of being “very rich”: 1-5 points, a higher score means more important	28,184	3.702	1.195	1	5
Age	Age	Age of respondents	28,184	47.838	15.843	18	96
Educational background	Edu	Have you received a higher education: College degree or above is 1; other education level is 0	28,184	0.624	0.484	0	1
Urban and rural area	City	Have urban household registration = 1; rural household registration = 0	28,184	0.737	0.44	0	1

**TABLE 2:** PsyCap, SocCap, and Happi.

Value of variable	PsyCap		SocCap		Happi	
	Frequency	Percentage	Frequency	Percentage	Frequency	Percentage
0			100	0.35	236	0.84
1	534	1.89	97	0.34	172	0.61
2	882	3.13	179	0.64	264	0.94
3	5,458	19.37	575	2.04	595	2.11
4	8,702	30.88	551	1.96	563	2.00
5	12,608	44.73	5,943	21.09	4,592	16.29
6			2,911	10.33	2,274	8.07
7			3,717	13.19	3,016	10.70
8			8,022	28.46	7,128	25.29
9			1,647	5.84	2,221	7.88
10			4,442	15.76	7,123	25.27
Total	28,184	100.00	28,184	100.00	28,184	100.00

### Features of the Main Variables

The average values of happiness, psychological capital, and social capital are first discussed based on the basic attributes of residents (Table 3). First, for the comparison classification of happiness, the mean of happiness for female respondents was 7.50, which was higher than 7.46 for men. The average happiness of urban residents was 7.71 higher than the 7.39 of rural residents, which indicate that the overall happiness index of urban residents is higher than that of rural residents. The average happiness of residents with higher education backgrounds was 7.51, which was higher than the 7.42 for those without higher education. Second, for the classification and comparison of psychological capital, the mean of PsyCap of male residents was 4.14, slightly higher than the 4.13 for women. The PsyCap index of urban residents is slightly lower than that of rural residents. However, in terms of the classification of educational background, the PsyCap of residents with higher education backgrounds is lower than that of those without higher education. The PsyCap in the study used the key measurement index of future confidence, so the uneducated residents can be considered to be more confident in the future. Third, for the classified statistics of social capital, the mean of social capital for female residents was 7.16, which was higher than that of male residents. The index for rural residents is lower than that of urban residents. Significant differences in social capital are found among residents with different educational backgrounds. The average value of social capital of residents with higher education backgrounds was 7.23, which is significantly higher than the 6.97 for those without higher education. The social capital of this paper uses its core index, namely, the individual’s popularity relationship score; this means that higher education improves individuals’ capability to manage interpersonal relationships and obtain a wider range of networks and high-quality popularity.

**TABLE 3:** Mean analysis of the main variables.

Mean of variables	Male	Female	Urban	Rural	Higher education	No higher education
Happi	7.46	7.50	7.71	7.39	7.51	7.42
PsyCap	4.14	4.13	4.09	4.15	4.10	4.19
SocCap	7.11	7.16	7.17	7.12	7.23	6.97

### Regression Analyzes

As shown in Table 4, we first independently regressed the relationship between psychological capital and social capital on subjective happiness (Column 1 of Table 2). The regression result of PsyCap and social capital was significant at the 1% level, showing that improvements to PsyCap and social capital level can contribute to increased happiness. Column 2 shows the interaction between PsyCap and social capital, demonstrating that the impact of social capital on happiness is strengthened by PsyCap.

**TABLE 4:** Ordered probit regression.

	(1)	(2)	(3)
PsyCap	0.368*** (0.007)	0.24*** (0.021)	0.202*** (0.021)
SocCap	0.251*** (0.004)	0.173*** (0.013)	0.169*** (0.013)
Psych_Socia		0.019*** (0.003)	0.019*** (0.003)
Healt			0.11*** (0.006)
InCla			0.073*** (0.006)
Male			–0.055*** (0.013)
House			0.127*** (0.013)
Trust			0.097*** (0.013)
Money			0.018*** (0.005)
Age			0.005*** (0.000)
Edu			–0.053*** (0.015)
City			0.136*** (0.015)
cut1	0.456*** (0.04)	–0.047 (0.088)	0.484*** (0.095)
cut2	0.686*** (0.037)	0.181** (0.088)	0.717*** (0.094)
cut3	0.919*** (0.035)	0.412*** (0.087)	0.954*** (0.093)
cut4	1.259*** (0.034)	0.748*** (0.087)	1.298*** (0.093)
cut5	1.48*** (0.034)	0.967*** (0.087)	1.521*** (0.094)
cut6	2.411*** (0.034)	1.894*** (0.088)	2.465*** (0.094)
cut7	2.707*** (0.035)	2.189*** (0.088)	2.767*** (0.095)
cut8	3.055*** (0.035)	2.538*** (0.089)	3.123*** (0.095)
cut9	3.842*** (0.037)	3.327*** (0.089)	3.926*** (0.095)
cut10	4.119*** (0.038)	3.605*** (0.089)	4.207*** (0.095)
Observations	28184	28184	28184
*P* value (LR test)	0.000	0.000	0.000
Pseudo R^2^	0.089	0.09	0.098

Standard errors (SEs) are in parentheses, ***p < 0.01, **p < 0.05, and *p < 0.1.

Column 3 illustrates the results by adding the relevant control variables. The regression coefficient of the health variable (Healt) was significantly positive, consistent with previous studies in this field. Thanks to the improvement of living standards in China, such as the continuous improvement of medical care, food quality, environment, and other conditions, the health level of residents has been continuously improving, corresponding with the increasing happiness among residents. The variable of local income class was significantly positive, indicating that personal income is positively correlated with subjective happiness. Interestingly, gender was a significantly negative predictor, indicating that, under the same conditions, it is easier for female residents to obtain happiness. One possible explanation for this is related to the profound changes in the survival and development environment of Chinese women. In the course of reform for over 40 years, the rights of Chinese women have made great progress. Women are no longer simply the traditional family role of “taking care of the husband and children,” and women’s rights in education, work, social activities, cultural, and political life have all grown significantly.

Property ownership also had a significant impact on happiness, in which owning property was a significant indicator of happiness. For those respondents without property ownership, some rent houses, and some live in places sponsored by employers. When conducting this survey, overall housing prices in China were rising, which also created disparities in wealth; this was particularly evident between those who own residences and those who do not, i.e., those who rent (Lin et al., 2012). Trust in strangers was significantly positively associated with happiness, suggesting that a greater willingness to believe strangers reflects a certain psychological quality of residents that is conducive to experiencing happiness. This psychological quality may be related to courage, generosity, cheerfulness, and other factors.

Attitudes toward money were significantly negatively related to happiness, indicating that a strong emphasis on money is not conducive to more happiness. While there was a significant relationship between subjective happiness with age, it was numerically smaller compared with the other variables. China prides itself on the traditional logic of “standing up in society at the age of 30 years, living without a doubt at the age of 40 years, and clarifying one’s destiny at the age of 50 years.” Therefore, elderly residents generally have stable families, more mature and stable social capital, and also more mature and optimistic PsyCap, which all contribute to happiness. In addition, the substantial improvement of material living standards and the continuous enrichment of residents’ cultural living space are also conducive to the sense of gain and happiness in elderly residents.

Interestingly, the educational background was found to be significantly negatively related to happiness, indicating residents without higher education are more prone to happiness. This may be explained by the fact that residents without higher education may experience a relatively simple environment in employment and a small living circle. This is in contrast to complex working and living environments, which can instigate the anxiety and depression often found in highly-educated people. In contrast, the urban vs. rural results showed that urban residents are more likely to experience happiness than rural residents. The explanation for this may be that urban residents tend to have more convenient living facilities, more employment opportunities, better educational conditions, and better social security.

## Mediation Analyzes

In the basic regression analyzes, we found significant interactive effects between PsyCap and social capital, in which PsyCap strengthened the impact of social capital on happiness. In SEM analysis, we investigated whether there is an intermediary effect between PsyCap and happiness.

**FIGURE 1:**
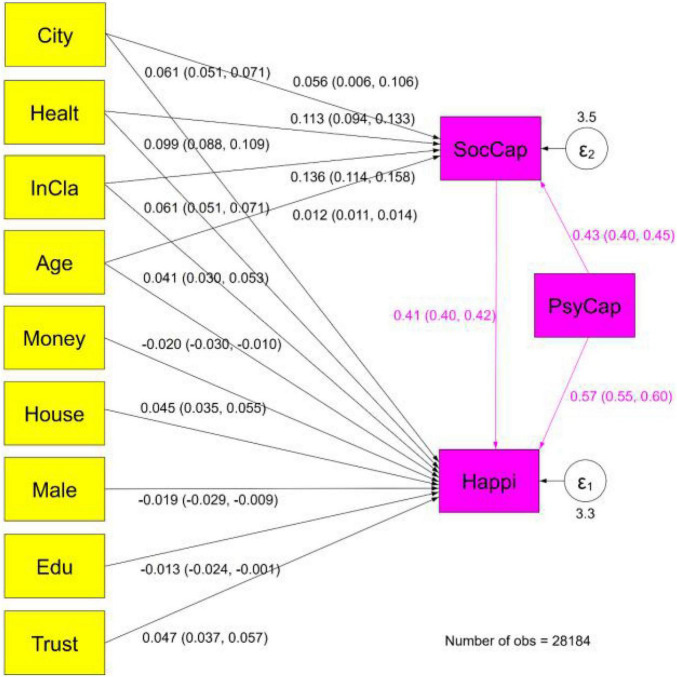
Mediation effect estimation results. Standardized parameter estimates and confidence intervals are in parentheses.

In the SEM model, we took social capital as the mediating variable, PsyCap as the direct variable, and happiness as the outcome variable; other covariant variables remain unchanged. We additionally constructed several models in which variables including gender, education level, trust, income class, and age were considered covariant variables of social capital. All models are shown in Figure 1.

The fitness evaluation indexes of the structural equation are shown in Table 5, and the estimated results are in Figure 1. According to the adaptation index of the structural equation, the model designed is reasonable and the estimated effect is robust.

**TABLE 5:** Fit statistic of the model.

Fit statistic	Value	Description
**Likelihood ratio**		
chi2_ms(5)	305.898	Model vs. saturated
*P* > chi2	0.000	
chi2_bs(23)	12822.301	Baseline vs. saturated
*P* > chi2	0.000	
**Population error**		
RMSEA	0.046	
90% CI, lower bound	0.042	
Upper bound	0.051	Root mean squared error of approximation
pclose	0.918	Probability RMSEA ≤ 0.05
**Information criteria**		
AIC	982407.5	Akaike’s information criterion
BIC	982572.4	Bayesian information criterion
Baseline comparison		
CFI	0.976	Comparative fit index
TLI	0.901	Tucker-Lewis index
**Size of residuals**		
SRMR	0.010	Standardized root mean squared residual
		

Figure 1 shows the direct, indirect, and total effects using the normalization coefficient. Estimates of social capital on happiness demonstrated a positive effect within the confidence interval, confirming that PsyCap had a positive effect on social capital. Furthermore, we found an indirect effect of PsyCap on happiness. Finally, we also found a total effect of PsyCap on happiness.

We then compared the effects of psychological capital and social capital on happiness. The direct, indirect, and total effects are reported in Table 6 using standardized coefficients. Since we utilized standardized coefficients, the effect sizes are directly comparable.

**TABLE 6:** Direct, indirect, and total effects.

Pathway	Direct effects	Indirect effects	Total effects
PsyCap→Happi	0.57***	0.18***	0.75***
SocCap→Happi	0.41***		0.41***

Standardization factors, ***p < 0.01, **p < 0.05, and *p < 0.1.

The analyzes show that the effect of PsyCap on happiness was greater than that of social capital. Therefore, psychological capital has more influence than social capital on the subjective happiness of Chinese residents.

## Heterogeneity Analysis

To analyze the role of PsyCap and social capital on happiness in detail, we investigated the following variables more closely: gender, urban/rural residence, and educational background. Estimated results are shown in Table 7. Columns (1) and (2) show that PsyCap and social capital have a greater influence coefficient on the happiness of male residents. However, the interaction term of PsyCap and social capital shows that PsyCap can better promote the impact of social capital on female residents’ happiness. Compared with the data in columns (3) and (4), the influence coefficient of PsyCap on urban residents is greater than that of rural residents. However, the social capital coefficient of rural residents is greater than that of urban residents. If compared with rural residents, urban residents gain more happiness because of PsyCap, and if compared with urban residents, rural residents gain additional benefits through social capital to promote happiness. At the same time, PsyCap can better promote the impact of the social capital of urban residents on happiness.

**TABLE 7:** Results of the heterogeneity analyzes.

	(1)	(2)	(3)	(4)	(5)	(6)
	
	Male	Female	Urban	Rural	Higher education	No higher education
PsyCap	0.227*** (0.030)	0.180*** (0.030)	0.246*** (0.047)	0.194*** (0.024)	0.153*** (0.027)	0.290*** (0.036)
SocCap	0.183*** (0.018)	0.156*** (0.018)	0.157*** (0.028)	0.173*** (0.014)	0.140*** (0.016)	0.225*** (0.022)
Psych_Socia	0.014*** (0.004)	0.023*** (0.004)	0.024*** (0.007)	0.017*** (0.003)	0.027*** (0.004)	0.004 (0.005)
Healt	0.110*** (0.008)	0.109*** (0.008)	0.082*** (0.012)	0.115*** (0.006)	0.100*** (0.007)	0.126*** (0.010)
InCla	0.068*** (0.009)	0.078*** (0.009)	0.058*** (0.014)	0.077*** (0.007)	0.064*** (0.008)	0.089*** (0.011)
House	0.150*** (0.019)	0.103*** (0.019)	0.106*** (0.025)	0.133*** (0.016)	0.117*** (0.017)	0.143*** (0.022)
Trust	0.108*** (0.018)	0.084*** (0.018)	0.073*** (0.026)	0.107*** (0.015)	0.096*** (0.016)	0.101*** (0.021)
Money	–0.022*** (0.008)	–0.013* (0.008)	–0.034*** (0.011)	–0.013** (0.006)	–0.015** (0.007)	–0.021** (0.009)
Age	0.006*** (0.001)	0.004*** (0.001)	0.007*** (0.001)	0.004*** (0.001)	0.007*** (0.001)	0.002*** (0.001)
Edu	–0.054** (0.022)	–0.054** (0.021)	–0.017 (0.031)	–0.053*** (0.018)		
City	0.148*** (0.021)	0.126*** (0.021)			0.159*** (0.017)	0.101*** (0.028)
Male			–0.031 (0.025)	–0.063*** (0.015)	–0.041** (0.016)	–0.082*** (0.021)
cut1	0.649*** (0.135)	0.384*** (0.133)	0.292 (0.207)	0.49*** (0.107)	0.409*** (0.12)	0.775*** (0.158)
cut2	0.923*** (0.133)	0.573*** (0.132)	0.550*** (0.203)	0.720*** (0.106)	0.647*** (0.119)	1.001*** (0.157)
cut3	1.130*** (0.133)	0.842*** (0.131)	0.775*** (0.202)	0.959*** (0.106)	0.87*** (0.118)	1.261*** (0.156)
cut4	1.454*** (0.133)	1.207*** (0.131)	1.137*** (0.202)	1.302*** (0.106)	1.210*** (0.118)	1.613*** (0.157)
cut5	1.689*** (0.133)	1.419*** (0.131)	1.397*** (0.202)	1.519*** (0.106)	1.45*** (0.118)	1.811*** (0.157)
cut6	2.612*** (0.134)	2.385*** (0.132)	2.325*** (0.204)	2.472*** (0.107)	2.38*** (0.119)	2.778*** (0.158)
cut7	2.932*** (0.135)	2.669*** (0.133)	2.671*** (0.204)	2.764*** (0.107)	2.685*** (0.120)	3.077*** (0.159)
cut8	3.304*** (0.135)	3.010*** (0.133)	3.120*** (0.205)	3.092*** (0.107)	3.040*** (0.120)	3.437*** (0.159)
cut9	4.110*** (0.136)	3.810*** (0.134)	4.090*** (0.206)	3.838*** (0.108)	3.855*** (0.121)	4.222*** (0.160)
cut10	4.387*** (0.136)	4.097*** (0.134)	4.470*** (0.206)	4.086*** (0.108)	4.144*** (0.121)	4.492*** (0.160)
Observations	14,101	14,083	20,781	7,403	17,585	10,599
*P* value (LR test)	0.000	0.000	0.000	0.000	0.000	0.000
Pseudo R^2^	0.095	0.101	0.096	0.105	0.101	0.093

Standard errors (SEs) are in parentheses, ***p < 0.01, **p < 0.05, and *p < 0.1.

We further found that the variable in urban residents was not significant. Specifically, we found that rural-dwelling men were less likely to be happy than rural women. Moreover, those rural residents with higher education backgrounds were less likely to be happy. In fact, most of the residents with higher education backgrounds in China choose to work in urban areas. College students who work and live in rural areas do not significantly account for this large section, because they have better knowledge and a broader vision, which, if failed to be applied, would decrease the sense of achievement. This phenomenon may result in cases of “high degree, low happiness” in rural areas. Columns (5) and (6) show the influence coefficient of PsyCap and social capital on happiness, demonstrating that these two factors have a greater influence on residents with higher education backgrounds than on residents without higher education. Interestingly, the interaction of psychological and social capital was not significant in the uneducated sample. This means that PsyCap does not significantly promote the effect of social capital on happiness in this particular group.

## Discussion

Although this topic is an important research direction in this field, our results represent only the beginning of such investigations into the mechanisms and pathways of the influence of psychological capital and social capital on residents’ mental health and happiness, suggesting many avenues for future work. In particular, it would be helpful to analyze related influencing factors.

## Conclusion

In this study, both psychological capital (PsyCap) and social capital were found to have a significant positive relationship with happiness in Chinese residents. PsyCap had a direct and indirect impact on happiness, while social capital mediated the effect of psychological capital on happiness.

## Data Availability Statement

The original contributions presented in this study are included in the article/supplementary material, further inquiries can be directed to the corresponding author.

## Author Contributions

XZ: writing. QL: analysis. SZ and TL: data curation. BH: supervision. All authors contributed to the article and approved the submitted version.

## Conflict of Interest

The authors declare that the research was conducted in the absence of any commercial or financial relationships that could be construed as a potential conflict of interest.

## Publisher’s Note

All claims expressed in this article are solely those of the authors and do not necessarily represent those of their affiliated organizations, or those of the publisher, the editors and the reviewers. Any product that may be evaluated in this article, or claim that may be made by its manufacturer, is not guaranteed or endorsed by the publisher.
